# The *Kanker Nazorg Wijzer* (Cancer Aftercare Guide) protocol: the systematic development of a web-based computer tailored intervention providing psychosocial and lifestyle support for cancer survivors

**DOI:** 10.1186/s12885-015-1588-z

**Published:** 2015-08-11

**Authors:** Roy A Willems, Catherine AW Bolman, Ilse Mesters, Iris M Kanera, Audrey AJM Beaulen, Lilian Lechner

**Affiliations:** 1 Faculty of Psychology and Educational Sciences, Open University of the Netherlands, P.O. Box 2960, 6401 DL Heerlen, The Netherlands; 2 CAPHRI School for Public Health and Primary Care, Maastricht University, P.O. Box 616, 6200 MD Maastricht, The Netherlands

**Keywords:** Cancer survivorship, eHealth, Quality of life, Psychosocial well-being, Lifestyle, Intervention Mapping, Computer tailoring, Problem-solving, Cognitive behavioral therapy

## Abstract

**Background:**

After primary treatment, many cancer survivors experience psychosocial, physical, and lifestyle problems. To address these issues, we developed a web-based computer tailored intervention, the *Kanker Nazorg Wijzer* (Cancer Aftercare Guide), aimed at providing psychosocial and lifestyle support for cancer survivors. The purpose of this article is to describe the systematic development and the study design for evaluation of this theory and empirical based intervention.

**Methods/design:**

For the development of the intervention, the steps of the Intervention Mapping protocol were followed. A needs assessment was performed consisting of a literature study, focus group interviews, and a survey study to get more insight into cancer survivors’ health issues. This resulted in seven problem areas that were addressed in the intervention: cancer-related fatigue, return to work, anxiety and depression, social relationships and intimacy, physical activity, diet, and smoking. To address these problem areas, the principles of problem-solving therapy and cognitive behavioral therapy are used. At the start of the intervention, participants have to fill in a screening questionnaire. Based on their answers, participants receive tailored advice about which problem areas deserve their attention. Participants were recruited from November 2013 through June 2014 by hospital staff from 21 hospitals in the Netherlands. Patients were selected either during follow-up visits to the hospital or from reviews of the patients’ files. The effectiveness of the intervention is being tested in a randomized controlled trial consisting of an intervention group (n = 231) and waiting list control group (n = 231) with a baseline measurement and follow-up measurements at 3, 6, and 12 months.

**Discussion:**

Using the Intervention Mapping protocol resulted in a theory and evidence-based intervention providing tailored advice to cancer survivors on how to cope with psychosocial and lifestyle issues after primary treatment.

**Trial registration:**

Dutch Trial Register NTR3375

## Background

With advances in cancer detection and treatment and an aging population the number of cancer survivors is increasing significantly [1]. It is well-known that survivors face a variety of difficulties and challenges after treatment, such as anxiety, depression, fear of recurrence, fatigue, pain, physical and cognitive limitations, difficulties with employment, and sexual dysfunctions [2–9]. These issues can have a negative impact on quality of life [8, 9] and may continue long after treatment has ended [10]. Cancer patients experience a peak level of distress within the first year after treatment, which might be partially explained by loss of security associated with being in treatment and loss of regular contact with health professionals [11]. Moreover, having a healthy lifestyle expedites recovery and, therefore, is of special importance for survivors. However, many survivors do not have a healthier lifestyle than people without a history of cancer [12]: more than half are overweight, less than half comply with physical activity recommendations, only one fifth adheres to fruit and vegetable recommendations, and one in ten smokes [13–16].

More than half of the survivors report having unmet information and support needs on how to deal with issues such as emotional and social support, fear and stress, and treatment and follow-up care [17–20]. Concerning lifestyle, survivors express a need for information and support regarding increasing exercise, improving diet, and smoking cessation [20–22]. To improve the aftercare for cancer survivors, a national guideline for cancer survivorship care was developed in the Netherlands [23]. This guideline describes the importance of a broad programmatic approach for oncology aftercare in which self-management should be stimulated.

The Internet has become a key source for health-related information for cancer survivors [24–26] and has the potential to fill an important gap in cancer care [27]. A great advantage of web-based interventions is that they can reach many patients at once and are accessible anytime and anywhere [28]. However, due to the broad variety of difficulties experienced and the different characteristics of the survivors, it is challenging to provide individually relevant information and support [20]. By means of computer tailoring, information and support can be provided that is adapted to the individual’s needs and characteristics, while still reaching large groups.

To provide cancer survivors personalized information and support and stimulating self-management during life after cancer, we developed the web-based computer tailored intervention the *Kanker Nazorg Wijzer* (Cancer Aftercare Guide; KNW). To increase the likelihood of reaching intervention effectiveness, the Intervention Mapping (IM) protocol was used [29]. This is a systematic, theoretical and empirical-based approach for intervention development. In this article, the development of the KNW according to the steps of IM and the evaluation of the intervention’s effectiveness is described.

## Methods/design

The IM protocol consists of six steps [29]: (1) a needs assessment of the study population, (2) specification of performance objectives and crossing them with relevant determinants into change objectives, (3) selecting theory-informed intervention methods and practical applications to change the determinants of the health behavior, (4) producing and pretesting program materials, (5) planning program adoption and implementation, and (6) planning for evaluation.

### Step 1: Needs assessment

In the needs assessment, the health problem and its impact on the quality of life of the at-risk group is assessed [29]. Understanding cancer survivors’ experienced problems and information and support needs is a crucial step in designing interventions that meet survivors’ needs [30]. Since it was clear that an overall problem among cancer survivors is a reduced quality of life [8, 9], the following program goal was stated: At six and twelve months after the start of the KNW program, cancer survivors will report an increased quality of life. The needs assessment aimed to disclose which problem areas should be addressed to achieve this goal.

We conducted a needs assessment consisting of a literature study, focus group interviews, and a survey. The literature provided an overview of cancer survivors’ health-related problems. Anxiety and depression [31], fear of recurrence [3], fatigue [4], sleep problems [32], difficulties concerning return to work [5], pain [33], and sexual dysfunction [34, 35] are frequently identified problems. Furthermore, a healthy lifestyle is associated with positive health outcomes in cancer survivors, while unhealthy lifestyle behaviors may lead to the development of other chronic diseases, new primary tumors, and cancer recurrence [36–39]. Unfortunately, a large proportion of cancer survivors do not adhere to recommendations concerning physical activity, dietary, and smoking behavior [13, 40].

Then, we conducted six focus group interviews with 33 cancer survivors using a predefined protocol [41]. The topics discussed included experienced problems during survivorship and aftercare needs. Most survivors indicated that they did not know what to expect after treatment or how to cope with their experienced problems. Commonly indicated problems included pain, fear of recurrence, fatigue, concentration problems, insomnia, sadness, insecurity, dealing with social relationships, and work related problems. Many survivors reported difficulties in adhering to physical activity and diet recommendations. However, physical activity was seen as an important contributor to recovery. Furthermore, many survivors indicated that they did not always know where they could get aftercare or that the aftercare was not easy accessible. Most survivors expressed the need for more attention from the hospital staff to their psychological, physical, and lifestyle issues. Moreover, the information provided by hospitals concerning aftercare possibilities was described as insufficient. The information on the Internet was described as cluttered and bulky.

Finally the prevalence and correlates of unmet information and support needs and healthy lifestyle behaviors were investigated in a survey conducted among 255 cancer survivors within the first year after their primary treatment [20, 42]. The results indicated that almost two-thirds of the survivors reported having unmet needs. Frequently cited unmet needs concerned emotional and social support, help to deal with fear of recurrence, management of healthcare and complications, up-to-date information, management of return to work, increasing exercise, and help to quit smoking. While help to eat healthier was not a frequently mentioned unmet need [20], adherence to fruit and vegetable recommendations was poor [42]. High education, having breast cancer, participation in support programs, low quality of life, high levels of anxiety, and a more negative adjustment to cancer were associated with having more unmet needs in general [20]. Self-efficacy, attitude, and intention were the strongest correlates of lifestyle behaviors [42].

### Step 2: Matrices of change objectives

Step 2 provides the foundation of the intervention by specifying what will change as a result of the intervention [29]. For this purpose, performance objectives (POs) are formulated. These are statements of what the program participants need to do to perform the intended health-promoting behavior. Then, important and changeable determinants for the POs are selected. This is necessary for creating change objectives (COs). COs specify what changes in the determinants are needed to make the attainment of the POs most likely.

To specify POs, it needs to be clear what the program outcome should be (i.e. what the program aims to achieve). Based on the needs assessment, the focus of the program was set to significantly reduce experienced problems in seven areas, namely (1) cancer related fatigue, (2) difficulties concerning return to work, (3) anxiety and depression, (4) social relationship and intimacy issues, (5) a lack of physical activity, (6) a lack of healthy food intake, and (7) difficulties in preparing or maintaining smoking cessation. By effectively managing these problems, improved outcomes in these problem areas are expected, ultimately resulting in a better quality of life.

Several POs were formulated for each problem area. An example of a PO for the program outcome “Reduce cancer-related fatigue” is “Say ‘no’ to a request when it is too much to handle” (see Table 1). Then, the most important and changeable behavioral determinants of the POs were selected from theory and literature. The most relevant determinants differed for each problem area. For example, relevant determinants for reducing cancer-related fatigue included knowledge, awareness, attitude, skills, self-efficacy, perceived behavior of others, and outcome expectations. Relevant determinants of engagement in sufficient physical activity included attitude, self-efficacy, social support, and perceived barriers. Next, COs were stated. Examples of COs for the PO “Say ‘no’ to a request when it is too much to handle” were “Describe steps to undertake to effectively say ‘no’ to others” (knowledge) and “See fellow survivors acknowledging the importance of saying ‘no’ to others” (perceived behavior of others) (see Table 2).

**Table 1:** Performance objectives for the program outcome “Reduce cancer-related fatigue”

**PO 1**	**Manage daily tasks efficiently**
PO 1.1	Alternate mental and physical activities
PO 1.2	Take small moments of rest divided over the day
PO 1.3	Take adequate measures so not to exceed personal limits
PO 1.4	Say “no” to a request when it is too much to handle
PO 1.5	Make a structured plan of daily activities
**PO 2**	**Turn non-helpful thoughts about fatigue into helpful thoughts**
PO 2.1	Recognize common non-helpful thoughts about fatigue
PO 2.2	Identify personal non-helpful thoughts
PO 2.3	Generate helpful thoughts
PO 2.4	Replace non-helpful thoughts with helpful thoughts
PO 2.5	Implement personal strategies to cope with rumination
PO 2.6	Use relaxation or mindfulness techniques
**PO 3**	**Take sleep hygiene measures**
PO 3.1	Identify the type of sleeping problem one is experiencing
PO 3.2	Go to bed and get out of bed at set times every day of the week
PO 3.3	Take care of optimal sleeping conditions
PO 3.4	Identify behaviors that interfere with sleep and replace these with helpful behaviors
PO 3.5	Use relaxation or mindfulness techniques

**Table 2:** Matrix of change objectives for the performance objective “Manage daily tasks efficiently”

*Reduce cancer-related fatigue*	Knowledge	Awareness	Attitude	Skills and Self-Efficacy	Perceived Behavior of Others	Outcome Expectations
PO.1. Manage daily tasks efficiently		Aw.1. Become aware of planning and structure of own daily activities	At.1. Feel positive about reorganizing daily activities	SSE.1. Feel confident about managing daily activities	PBO.1. See fellow survivors acknowledging the importance managing daily activities	OE.1. Expect that managing daily tasks efficiently can reduce feelings of fatigue
PO.1.1. Alternate mental and physical activities	K.1a. Describe the importance of alternating mental and physical activities	Aw.2. Become aware whether mental and physical activities are alternated in own daily scheme				OE.2. Expect that alternating mental and physical activities can reduce experiences of fatigue
	K.1b. Recall advice on alternating activities					
PO.1.2. Take small moments of rest divided over the day	K.2a. Describe the importance of taking small moments of rest	Aw.3. Become aware whether daily rest is divided in small moments over the day				OE.3. Expect that taking small moments of rest divided over the day can reduce experiences of fatigue
	K.2b. Recall advice on taking rest					
PO.1.3. Take adequate measures to not exceed personal limits	K.3a. Recall possible signals of exceeding personal limits		At.2. Feel positive about guarding personal boundaries	SSE.2. Feel confident about recognizing signals and taking adequate measures	PBO.2. See fellow survivors acknowledging the importance of not exceeding personal limits	OE.4. Expect that taking adequate measures when exceeding limits can reduce experiences of fatigue
	K.3b. Recall effective measures when exceeding limits					
PO.1.4. Say “no” to a request when it is too much to handle	K.4. Describe steps to undertake to effectively say “no” to others		At.3. Feel positive about saying “no” to others	SSE.3. Feel confident about saying “no” to others	PBO.3. See fellow survivors acknowledging the importance of saying “no” to others	OE.5. Expect that others generally accept when receiving “no” to a request
PO.1.5. Make a structured plan of daily activities	K.5. Summarize advice on making a structured plan			SSE.4. Demonstrate ability of making an efficient plan by incorporating advice in the new plan		OE.6. Expect that making a new plan will help in dealing with fatigue

### Step 3: Selecting theoretical methods and practical applications

In this step theoretical methods and practical applications for achieving the COs and POs are selected [29]. A theoretical method is a technique or process for influencing change in the determinants of the targeted behavior. A practical application is a specific technique for practical use of a theoretical method. For example, by means of self-monitoring of behavior (method) we aimed to change cancer survivors’ awareness of how they scheduled their daily activities (determinant) by encouraging them to register their daily activities for five to seven days (practical application) (see Table 3). Methods and applications were derived from literature, focus group interviews, and existing interventions (see Step 4, Reviewing available materials).

**Table 3:** Methods and applications to change the determinants of the performance objective “Manage daily tasks efficiently”

Determinant	Theoretical methods	Practical applications
Knowledge	Chunking	Advice provided is divided in several topics and is summarized when participants make their own planning.
	Elaboration	After providing advice, personally relevant messages encourage participants to incorporate this advice with their situation.
	Cues	Cues are provided that help saying “no” to a request and to recognize when personal limits are exceeded.
Awareness	Consciousness raising	Cancer survivors are encouraged to register their daily activities for five to seven days. After registration, survivors are given advice on effectively planning their day, asked to compare their plan with the advice received, and encouraged to adjust their plan to meet this advice.
	Self-monitoring of behavior	
Attitude	Arguments	Cancer survivors are given arguments why efficiently planning daily activities is beneficial for reducing fatigue, why guarding personal boundaries is important, and why saying “no” to some requests is important.
Skills and Self-Efficacy	Active learning	Cancer survivors are encouraged to make their own weekly plan using the advice given.
	Action planning	Cancer survivors are encouraged to make a list of personal signals indicating that limits are exceeded and select adequate measures for each signal.
		Cancer survivors are encouraged to make their own action plan for when they are in a situation in which they want to say “no” to a request.
Perceived Behavior of Others	Modeling	Cancer survivors are provided with narratives of other survivors who are further along in their recovery process. In these narratives the importance and effectiveness of planning daily activities, setting personal boundaries, and saying “no” to others is explained.
Outcome Expectations	Persuasive communication	By providing information from different sources (e.g., peers) on managing daily activities and by making assignments, cancer survivors are encouraged to expect that fatigue can be dealt with when taking adequate measures.
	Active learning	
	Modeling	

Several methods were used in the KNW such as feedback, personalizing risk, consciousness raising, belief selection, modeling, active learning, persuasive communication, argumentation, goal setting, action planning, and implementation intentions. Two methods formed the core of the KNW: *tailoring* and *skills training for self-management*. These two methods were used throughout the entire intervention and were combined with the other methods to change the determinants of the targeted behaviors.

#### Tailoring

Tailoring is a technique in which information is provided that is adapted to the personal characteristics circumstances, beliefs, motivations, and behavior of the receiver [43, 44]. Thus, by means of tailoring, personalized advice can be provided that suits the cancer survivors’ needs. Overall, tailoring is proven to be an effective technique in health promotion and communication [43, 45–48]. Since the information is personalized, less redundant information is provided, attention is increased, information is more thoughtfully processed, and behavior change or maintenance is better facilitated [43, 44, 49, 50]. The KNW starts with a screening questionnaire that enables tailoring. Based on their answers, participants receive feedback about which of the seven problem areas deserve their attention (see also Step 4, Screening questionnaire). When selecting a problem area that the participant wants to work on, the information on that problem is tailored further, eventually resulting in a personalized action plan.

#### Skills training for self-management

Self-management is an iterative process that comprises observation of one’s behavior (monitoring) making judgments of behavior based on the observation (evaluation), setting goals, and choosing and applying strategies to achieve these goals (action) [29, 51, 52]. The principles of problem-solving therapy (PST) [53, 54] and cognitive behavioral therapy (CBT) [55] were used as applications to increase cancer survivors’ self-management skills. PST and CBT for cancer patients and survivors have been found effective for, amongst others, improving symptom management [56, 57], mental health and quality of life [58, 59], dealing with uncertainty [57], fatigue [60–62] and insomnia [63], and reducing psychological distress [64, 65].

PST comprises five steps in which the patient (1) needs to adopt a positive attitude towards facing the problem, (2) defines what the problem exactly is, (3) makes a list of alternatives to tackle the problem, (4) predicts the benefits and consequences of each alternative, and (5) implements the best alternative in daily life and evaluates the result [53]. In the KNW, each problem area is addressed following the structure of PST; that is, identifying the problem and selecting a goal, getting informed of different solutions, making a personalized action plan, and trying out the action plan and evaluating the progress.

The basic principles of CBT are covered by providing psycho-education and giving assignments such as monitoring behavior or thoughts, challenging dysfunctional cognitions, and encouraging patients to set new goals. In addition, elements were used from a treatment protocol proven effective for treating cancer-related fatigue among cancer survivors [55]. The protocol links six factors to fatigue: (1) poor coping with cancer, (2) fear of cancer recurrence, (3) dysfunctional cognitions, (4) dysregulation of sleep, (5) dysregulation of activity, and (6) low social support. All these factors are addressed in the KNW.

### Step 4: Producing program components and materials

With the end products of the previous steps, the program components and materials were produced. This included describing the program scope and sequence, preparing design documents, reviewing available materials, and developing and testing the program materials [29].

#### Scope and sequence

The KNW (http://www.kankernazorgwijzer.nl) covers seven self-management training modules. The modules Fatigue, Return to Work, Mood (i.e. anxiety and depression), and Relationships mainly cover psychosocial and mental health related issues, while the modules Physical Activity, Diet, and Smoking cover lifestyle-related issues. The modules are interrelated. For example, within the Fatigue module, participants receive the advice to also visit the Physical Activity module if there is an indication that the participant is getting too little physical activity. As discussed in Step 3, the sequence within the modules is based on PST [53]. In general, the modules consist of four components divided over two sessions. In the first session, participants identify their problem, select a goal and receive psycho-education and assignments on how to deal with their problem, and personalize their goal through action plans. After thirty days, participants are invited for a second session in which they can evaluate the progress of their goal. If successful, participants are encouraged to maintain their behavior. Otherwise, participants are encouraged to try again, try another solution, or adjust their goal and receive additional advice on how to deal with difficult situations. Furthermore, all modules provide links to other relevant and reliable websites.

**Fig. 1:**
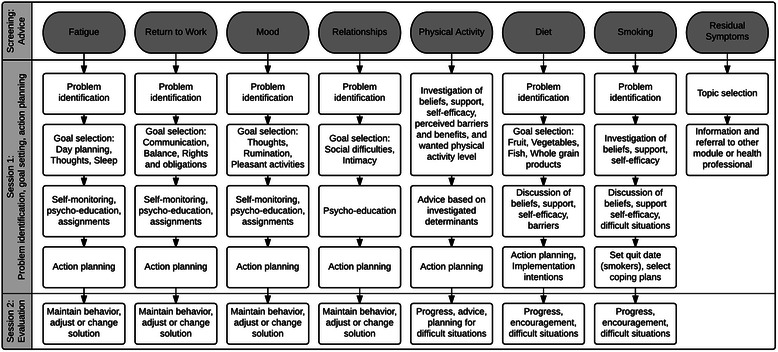
Overview of the scope and sequence of the modules

Participants of the focus groups (see Step 1) expressed the need to be informed about commonly experienced complaints after cancer treatment. Therefore, an additional module covering residual symptoms from cancer treatment was added to the KNW. In this module, general information is given on the most common physical complaints experienced after primary treatment, tips are given on how to deal with these symptoms, and advice is given to seek medical assistance for more information or help. For an overview of the scope and sequence of all modules, see Fig. 1.

To keep participants involved in the program several types of e-mails were sent. First, participants received reminder e-mails when they completed the screening questionnaire but did not visit any of the modules. Second, participants received an e-mail to invite them to the second session of a module. Third, participants received a postcard in spring wishing them Happy Easter and an eCard around the Holidays wishing them Happy Holidays. Fourth, monthly news items were placed on the website in which professionals from different fields talk about cancer recovery (see Step 4, Video material). Participants received an invitation e-mail to see the latest news item.

#### Suggestions from the target group

**Fig. 2:**
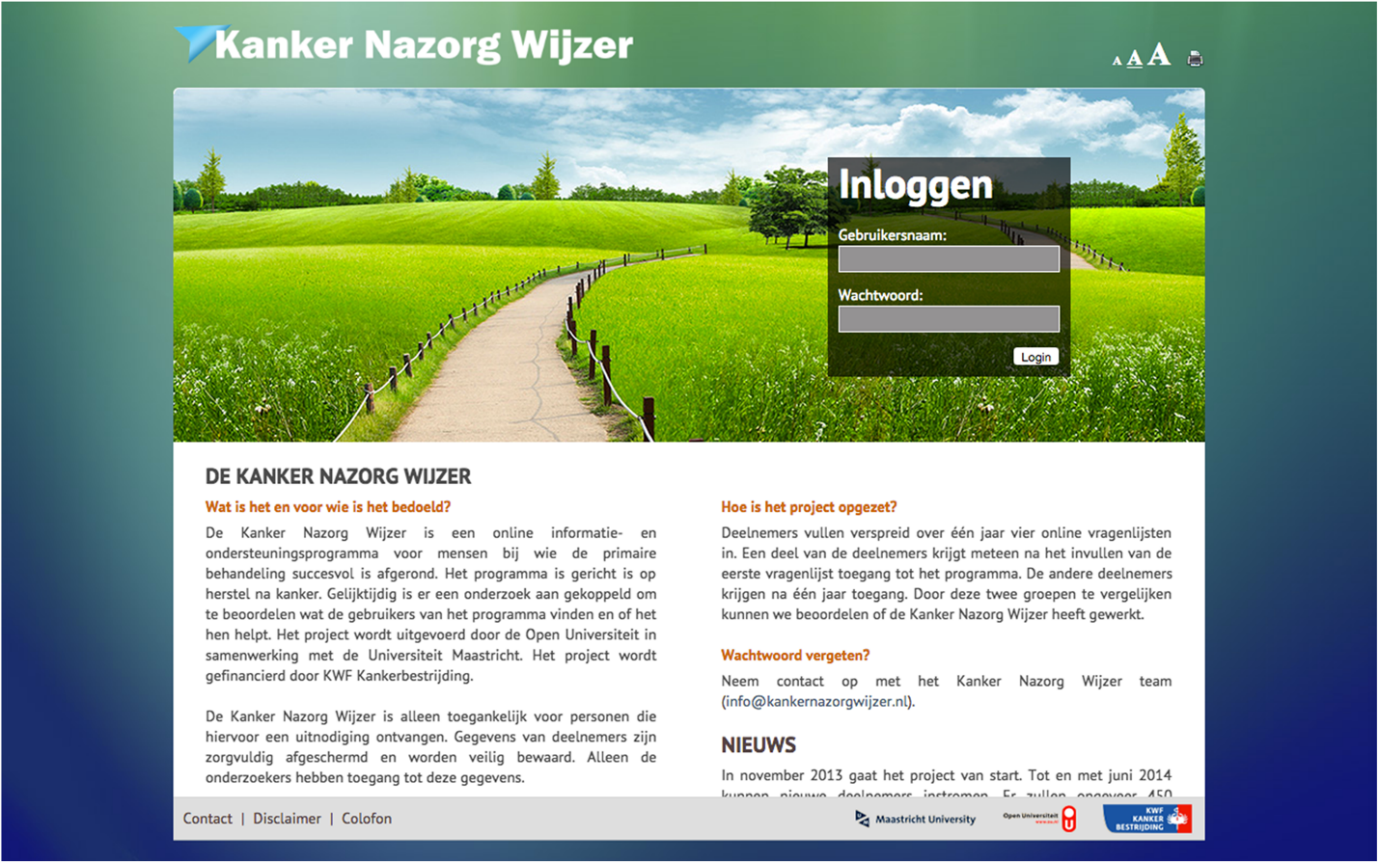
The appearance of the KNW

During the focus group interviews (see Step 1), the preferences for the look and feel of the future program were discussed. First, survivors suggested messages to be framed positively and that the program should have a calm and friendly appearance (see Fig. 2). Second, survivors indicated that they preferred an open and unrestrictive program. Therefore, the KNW is programmed in such a way that users can choose which modules of the intervention they want to follow, even if they get the advice that they are doing well in this area. Third, survivors mentioned that the intervention should be easy to use. Therefore, a website with clear and distinctive buttons was designed with an emphasis on preventing an overload of information. Finally, it was suggested that the written information should be supported with video material. We adhered to this by providing informational videos from professionals from different fields. Also, there was a high demand for videos of fellow survivors, who were further into their recovery process, discussing their experiences of their life after cancer treatment. Therefore, we interviewed eight former patients discussing their cancer recovery and giving advice on how to deal with certain issues (see Step 4, Video material).

#### Reviewing available materials

Before developing the program materials, available program materials of others were reviewed for a possible match with the COs, methods, and applications of the KNW [29]. There were some computer tailored interventions from which elements were usable for the modules of the KNW. For the Physical Activity module, we shortened and adjusted the Active Plus intervention [66–69] to meet the characteristics of our target group. We also used elements from computer tailored interventions on smoking cessation [70–72] and nutrition [73–77]. As mentioned in Step 3, the Fatigue module was based on a protocol for treating cancer-related fatigue [55].

#### The intervention

##### Screening questionnaire.

**Fig. 3:**
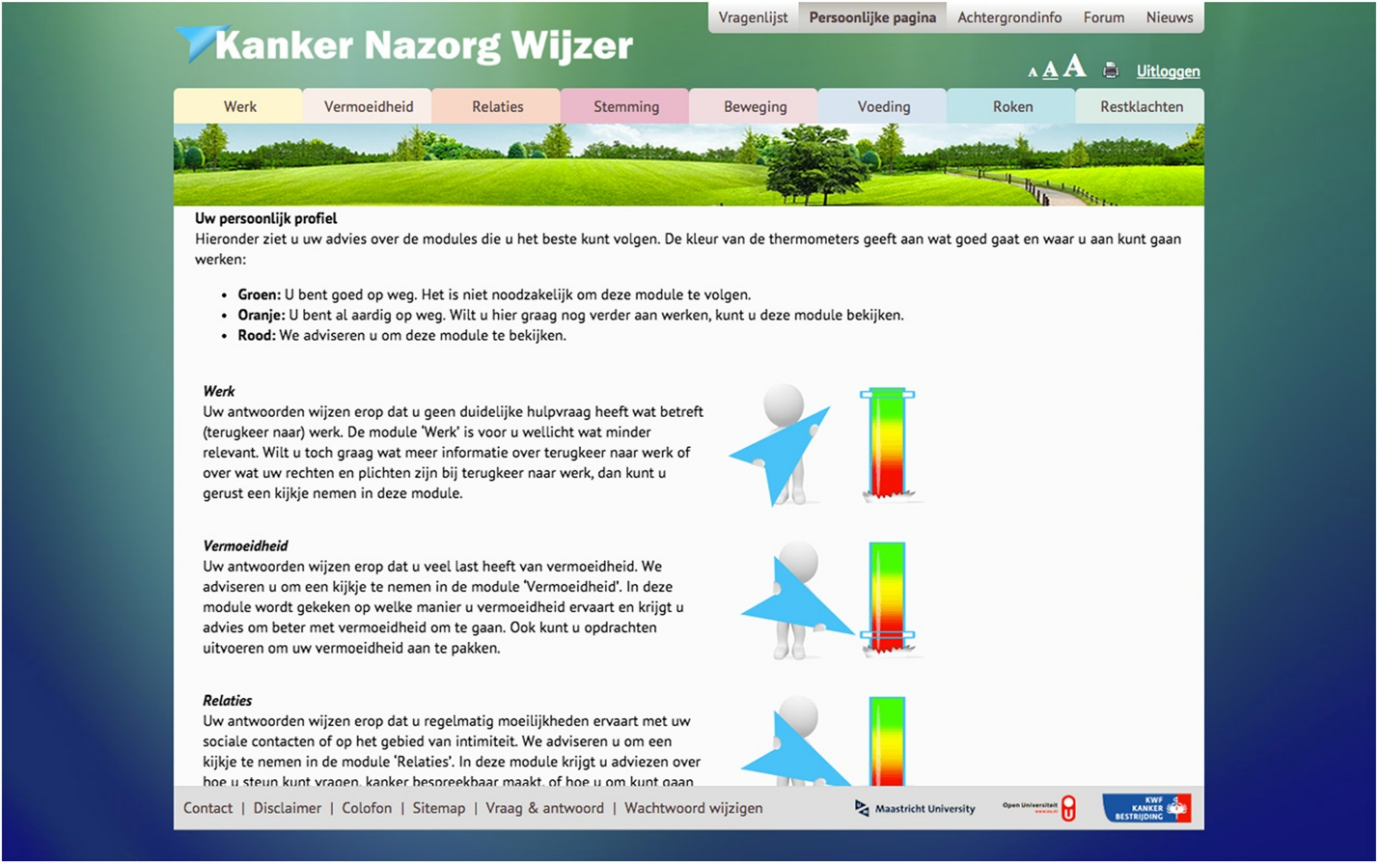
After screening, participants are advised which module deserve their attention

The KNW starts with a screening questionnaire measuring several concepts, including fatigue, work limitations, psychological distress, social support, physical activity, food intake, and smoking behavior (see Step 6, Measurements). Based on their answers, participants receive personal advice about which modules deserves their further attention. For this, a thermometer is used as visual aid (see Fig. 3). “Green” advice indicates that the participant is doing well in this area and visiting the corresponding module is not necessary. “Orange” advice indicates that the participant is doing reasonably well, but there still is room for improvement. “Red” advice indicates that the participant is strongly advised to visit the corresponding module.

#### Modules

##### Fatigue.

In the Fatigue module, cancer-related fatigue is addressed. Based on the answers of the screening questionnaire, participants receive a description of the type of fatigue they are most likely experiencing. Participants receive an improvement proposal, comprising the themes day plan, fatigue-related thoughts, sleeping behavior, feelings of anxiety or depression, relationships, and physical activity. When participants want to work on physical activity, relationships, or feelings of anxiety and depression, they are referred to the Mood, Relationships, and Physical Activity modules, respectively.

The theme “Day Plan” discusses the importance of a structured day plan. Participants are encouraged to monitor their daily activities for five to seven days. Then, psycho-education and assignments are given concerning planning activities and rest, not to exceed personal limits, and saying “no” to requests. Finally, participants are encouraged to make a weekly plan.

The theme “Thoughts About Fatigue” discusses beliefs concerning fatigue that are fatigue enhancing. Psycho-education and assignments are given on recognizing and identifying non-helpful thoughts. Participants are encouraged to register their own non-helpful thoughts for one week. Then, these thoughts are challenged by discussing their credibility and usefulness and advice is given on how to replace these thoughts with helpful thoughts. Furthermore, advice is given on how to deal with rumination. Finally, to deal with stress related to dysfunctional cognitions, information and assignments are given concerning relaxation and mindfulness.

The theme “A Good Night’s Sleep” discusses participants’ sleeping behavior. Participants are encouraged to monitor their sleep and wake times for one week. Then, psycho-education and assignments are given concerning types of sleeping problems, the importance of a consistent sleep-wake pattern, and sleeping hygiene. Also, information and assignments are given concerning relaxation and mindfulness.

##### Return to Work.

In the Return to Work module, difficulties and rights and obligations concerning returning to work are discussed. Based on the answers of the screening questionnaire, participants receive an overview of their indicated problems concerning return to work and are given the opportunity to further specify these problems. Then, participants select a goal that they want to achieve (e.g., learning to ask for help). Depending on the chosen goal, participants are advised to continue with one of the three themes: Communication, Balance, and Rights and Obligations.

The theme “Communication” discusses the preparation of difficult work-related conversations. Psycho-education and assignments are given on preparing work-related conversations with different persons, such as one’s occupational physician, supervisor, or colleague. Advice is given on, amongst others, how to indicate possibilities and limitations with regard to work tasks, asking for help, dealing with incomprehension from the manager or colleagues, or preparing a job application. Moreover, advice and assignments are given on how to increase feelings of confidence and decrease feelings of stress in difficult interactions.

The theme “Balance” focuses on finding a balance between the participants’ work abilities and their workload. Participants are encouraged to monitor for several workdays how much energy certain work-related tasks cost. Then, psycho-education and assignments are given concerning planning the workday, not to exceed personal limits, making adjustments at work, dealing with limited concentration and memory problems, relaxation, and thinking positively.

The theme “Rights and Obligations” provides information on cancer survivors’ rights and obligations concerning work with a long-term illness. Information is provided on topics such as re-integration unemployment, searching for a new job, social welfare payments, insurances, legal advice, or rights on facilities to perform one’s job properly, given the limitations caused by the disease or treatment.

##### Mood.

The Mood module focuses on feelings of anxiety and depression. More specifically the module discusses common anxiety and depression provoking thoughts and how to cope with these thoughts more effectively. Based on the answers of the screening questionnaire, participants receive feedback on their current state of anxiety, depression, and adjustment to cancer. When there is an indication that the participant is experiencing severe levels of psychological distress, a recommendation is given to visit one’s general practitioner to get a referral for help. In the module, participants first set a goal they want to achieve (e.g., to reduce feelings of sadness). Then, psycho-education and assignments are given concerning non-helpful or anxiety provoking thoughts, such as feelings of failure or fear of cancer recurrence. Participants are encouraged to monitor their inefficient thoughts for one week. Then, these thoughts are challenged by discussing their credibility and usefulness and advice is given on how to replace these thoughts with helpful thoughts. Furthermore, advice and assignments are given concerning planning pleasant activities, how to deal with rumination, and how to reduce feelings of anxiety or sadness by means of relaxation and mindfulness.

##### Relationships.

The Relationships module addresses coping with difficult social situations and intimacy problems. Difficult social situations are discussed, such as receiving inadequate help from others, social isolation, experiencing social pressure, and talking about having had cancer. Based on the answers of the screening questionnaire, participants receive an overview of social situations in which they wish change. After selecting such a social situation, psycho-education is given on how to constructively deal with this situation.

Concerning intimacy, psycho-education is given on discussing intimacy and sexuality with significant others and how to cope with sexuality with respect to physical and functional changes due to cancer treatment. Coping with physical and functional changes is tailored to gender. For example, men receive information on how to cope with issues such as erectile dysfunction or dry orgasms, while for women advice is given on how to cope with issues such as menopausal symptoms or vaginal problems.

##### Physical Activity.

In the Physical Activity module, participants are encouraged to increase their level of physical activity. Based on the answers of the screening questionnaire in combination with the Dutch physical activity guidelines, participants receive feedback on their own level of physical activity and to which extent it reaches the recommended level. Then, participants are encouraged to set a goal, for example, increasing physical activity during commuting, daily activities, leisure time, or sports. Subsequently, advice is given based on the participant’s beliefs about the pros and cons of exercising, perceived barriers and benefits, self-efficacy, and social support. Next, participants are encouraged to make a personal exercise plan. The module provides information on specific exercises and sport activities tailored to participant’s individual situation, physical limitations and social cognitive determinants.

##### Diet.

The Diet module focuses on increasing fruit, vegetables, whole grain bread, and fish consumption. Based on the answers of the screening questionnaire in combination with the Dutch nutritional guidelines, participants receive feedback on their dietary habits and the extent to which it reaches the recommended level. The module subsequently provides a standard, non-personalized overview of a healthy diet, including desirable and undesirable foods and an indication of the recommended servings. Afterwards participants are encouraged to set one or two goals, for example, eating two pieces of fruit per day or eating 200 grams of vegetables per day. Subsequently, dietary advice is given, personalized to the participant’s individual situation, experienced medical or treatment related problems, and the participant’s attitudes, self-efficacy, and social support toward performing the desired dietary behavior.

##### Smoking.

The Smoking module is developed for smokers to stimulate them to refrain from smoking and for former smokers to prevent relapse. Based on the answers of the screening questionnaire, participants’ current smoking behavior is discussed. Smokers are encouraged to quit and to set a quit date. Advice is given on how to anticipate risky situations for a lapse and how to deal with withdrawal symptoms. Smokers are encouraged to develop an individual coping plan to prepare their quit attempt and to deal with difficult moments to maintain abstinence. Former smokers also receive advice based on their individual situation and social cognitive determinants, aimed at the prevention of relapse. They are also encouraged to develop coping strategies to prevent relapse.

##### Residual Symptoms.

In the Residual Symptoms module, brief information is given about complaints, such as pain, lymphedema, osteoporosis, or neuropathy. If a certain topic is covered in one of the other modules, referral to the respective module is also given. Next to some basic tips on how to deal with these symptoms, participants are given advice to contact their physician or other health professional when they experience serious problems.

#### Other website elements

##### Personal page.

**Fig. 4:**
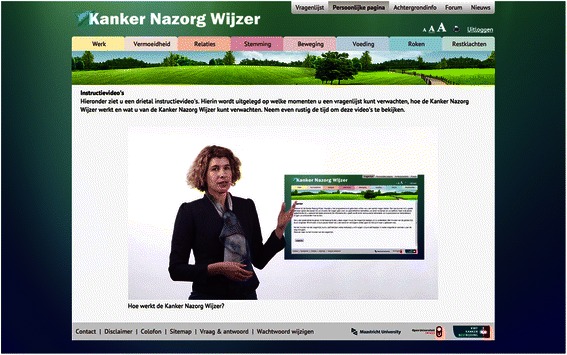
Example of an instructional video explaining how to use the KNW

On the Personal Page participants can find an overview of the personal advice they received from the screening questionnaire and the modules. Also the Personal Page contains a few instructional videos on how to use the KNW (see Fig. 4).

##### Video material.

The use of videos is an important component of the KNW. Text messages accompanied with video are more appreciated and better recalled than text messages only [78, 79]. There are four types of videos implemented. First, *instructional videos* explain what participants can expect from the KNW and how they should navigate the program. Second, *videos of fellow survivors* were included for which we interviewed eight cancer survivors who were further along in their recovery process and willing to share their experiences of their life after cancer treatment and give advice to deal with certain issues. Since men and women interviewed were from different age groups and recovering from different types of cancer, it is more likely that participants identify with one of these role models. Third, *videos of professionals* were included for which we interviewed a sexologist and two clinical psychologists. These professionals give psycho-education and advice from clinical practice. Fourth, with monthly *news items* participants were provided with extra information on specific areas*.* We interviewed professionals from different fields, talking about topics such as exercise, diet, return to work, anxiety and depression, lymphedema, and peer support groups. With these news items, we aim to keep the participants involved in the KNW by referring them to the module that is related to the topic discussed in the news item.

##### Forum.

The KNW has a forum where fellow survivors can meet and ask each other or members of the KNW team questions. Participants are kept anonymous and the KNW team monitors the forum to control for advice contradicting the advice given in the modules.

#### Pretesting and revising

The KNW was pretested among 13 cancer survivors. In general, the appearance and content of the KNW was highly appreciated. The modules were evaluated positively (*M* = 7.6, range = 1–10). Particularly, the videos of fellow survivors and professionals were highly rated (*M =* 8.0, range = 1–10). The mean scores for understanding, usefulness, reliability, applicability, completeness, and appearance of the KNW ranged from 3.3 to 3.7 (range = 1–4) and, therefore, were also highly appreciated. Some of the texts were evaluated as fairly long. Consequently, an editor reviewed and edited the text on readability and length. Furthermore, the tailored advice was evaluated to be of great value. While the tailored messages were computer generated, some participants initially thought that a person provided these messages. Although this demonstrated the power of tailoring, it also confused the participant when an answer did not fully match his or her expectations. Further, while the aim of the KNW is to stimulate participants to create their own solutions, some participants expected to receive personalized solutions to their problems. To address these issues, we included the previously mentioned instructional videos to explain how the KNW works and what participants can expect. After some final adjustments, the KNW was ready for implementation and effectiveness testing.

### Step 5: Adoption and implementation

In the fifth step, a plan for program adoption and implementation was developed [29] in the context of testing the effectiveness of the KNW in a randomized controlled trial (RCT). We created a network with representatives (e.g. department heads, oncologists, research nurses, and nurse practitioners) from several hospitals’ outpatient clinics in internal medicine, oncology, gynecology, urology, and the breast clinic. Forty-five hospitals in the Netherlands were contacted for assistance in the recruitment. Twenty-two hospitals agreed to participate, with 21 hospitals eventually participating in patient recruitment. Reasons for refusing participation included: hospital was already participating in other research projects, lack of time or excessive workload, too few staff members to recruit participants, or insufficient number of patients who met the inclusion criteria. Creating this network was a very time consuming process; it easily could take more than half a year from the moment of contacting a representative of a clinic until staff members began recruiting participants. Next, maintaining contact with multiple hospitals required good planning. To keep the staff members involved, we send out monthly newsletters with updates of the research project. Also, we regularly send thank-you cards. To conclude, timely planning of program adoption and implementation is essential.

### Step 6: Planning for evaluation

In the final step, a plan for the effect and process evaluation of the intervention was developed. While the effect evaluation describes the differences in outcomes between the participants who were and were not exposed to the KNW, the process evaluation aims to get insight into the use and appreciation of the intervention [29]. For the evaluation of the KNW, an RCT comparing the intervention group with a waiting list control group was conducted. The RCT is approved by the Medical Ethics Committee of the Atrium Medical Centre (NL41445.096.12) and is registered in the Dutch Trial Register (NTR3375).

#### Participants

Patients were eligible for participation if they were 18 years or older, they had been diagnosed with any cancer type, primary treatment (surgery, chemotherapy, and/or radiotherapy) had been completed successfully for at least 6 weeks but no more than 52 weeks, there was no sign of recurrence in the latest follow-up visit, they were able to read and speak Dutch, and there was no serious medical, psychiatric, or cognitive illness that would interfere with participation. Computer literacy was not an explicit inclusion criterion, since the hospital staff was not able to screen for this. We expected that patients who were not computer literate would not participate in the study.

#### Design and procedure

Staff members of 21 hospitals (see Step 5) recruited patients from November 2013 through June 2014. The recruitment period varied per hospital. Patients were selected either during follow-up visits to the hospital or from reviews of patients’ files. Oncologists, research nurses, and nurse practitioners from the outpatient clinics internal medicine, oncology, gynecology, urology, and breast clinics invited patients who met the inclusion criteria to participate by giving them an information package during a follow-up visit or sending the package to them following review of the patient’s files. The information package included: (1) a letter with trial information and a username and password for first login, (2) an informed consent form with return envelope, (3) an information brochure concerning Medical Research, (4) a short manual on how to use the KNW, and (5) a small card with contact details and space where participants could write down their new username and password. A reminder was send after two weeks. Patients who agreed to participate were requested to sign the informed consent form and return it to the Open University of the Netherlands. Patients who participated in the research but did not return the consent form were contacted to do so. If they did not return the informed consent after several reminders, they were excluded from evaluation.

After online registration participants were randomly assigned to either the intervention group or the waiting list control group. Both groups had to fill in a questionnaire at four time points: At baseline (T0), after three months (T1), after six months (T2), and after 12 months (T3). T1 aimed to measure possible mediating variables, while T2 and T3 aimed to measure the short- and long-term effectiveness of the intervention respectively. The intervention group had six months access to the KNW directly after baseline. The waiting list control group had access to the intervention after T3.

Several methods were used to increase the response rate. First, several automated e-mail reminders were sent for each measurement. Second, in the baseline measurement, participants could leave their telephone number so we could contact them concerning the research. When we noted that participants had not reacted to the e-mail reminders, we contacted them through telephone as a final reminder. Third, in the e-mail reminders participants were explained that they would receive a small token of appreciation at the end of the trial. That is, participants received a book voucher with a value of €10 for trial participation.

#### Measurements

The primary outcomes for the evaluation of the KNW comprise psychosocial well-being and lifestyle outcomes. Measuring psychosocial well-being comprised assessment of quality of life [80] psychological distress [81, 82], mental adjustment to cancer [83], fatigue [84], work limitations [85–87], and social support [88]. Measuring lifestyle comprised assessment of physical activity [89], food consumption [90], and smoking behavior [91]. Secondary outcomes included measures that were assumed to moderate or mediate the effects of the primary outcomes, such as resilience [92], self-control [93], personal control [94], problem-solving skills [95, 96], and several background characteristics (e.g., age, gender, education, employment status, and disease and treatment history). Finally, we measured cancer survivors’ unmet needs [97].

#### Power calculation

Sample size calculations were based on the outcomes of quality of life and psychological distress, since these were expected to be the most difficult to change. Calculations showed that 144 patients per group were required to compare means for these outcomes between groups with a power greater than .80, one sided with an alpha of 0.05. This was based on an expected effect size of .30 and, since recruitment would be through hospitals, a correction for multilevel analyses (intracluster correlation coefficient = .005, design effect = 1.15). With an expected dropout rate of 20% during the study, this meant 376 patients needed to be included at baseline. With 231 patients included in the intervention group and 231 patients in the waiting list control group at baseline, this target has been achieved.

## Discussion

The aim of this paper was to describe the systematic development and the study design for evaluation of the KNW, a web-based computer tailored intervention aimed at providing psychosocial and lifestyle support during life after cancer. The intervention aims to reduce cancer survivors’ experienced problems in seven areas: (1) cancer-related fatigue, (2) difficulties concerning return to work, (3) anxiety and depression, (4) relationships and intimacy issues, (5) a lack of physical activity, (6) a lack of healthy food intake, and (7) difficulties in preparing or maintaining smoking cessation. By reducing the experienced problems in these areas, it is expected that this ultimately will result in a higher quality of life. The intervention was developed using the IM protocol [29]. This protocol supports health promotion program planners in systematically developing a theory and evidence-based program, and, as a result, increasing the likelihood of its effectiveness.

Beside the systematic development, the KNW has several other strengths. First, since the KNW concerns a web-based intervention, it can reach many patients at once and is accessible anytime and anywhere [28]. Second, by means of tailoring, information is more personally relevant. Therefore, it is more likely that this information increases attention, is more thoughtfully processed, and facilitates behavior change or maintenance [43, 44, 49, 50]. Third, the use of video material to accompany the text also increases the likelihood that the information is remembered and recalled [78, 79]. Fourth, as universal methods, the KNW uses the principles of PST [54] and CBT [55] to stimulate cancer survivors to learn self-management techniques that they also can apply in other situations. Fifth, elements of the KNW are based on existing interventions that already have been proven effective. Sixth, by evaluating the KNW through an RCT, we will be able to draw conclusions of the intervention’s effectiveness.

However, there are also some weaknesses that should be mentioned. First, the intervention contains much written information. Since PST and CBT are quite extensive forms of therapy, it was a challenge to reduce the amount of information while still holding to the theoretical framework of these methods. Much information might particularly be a problem for people with low health literacy [98], since they might not be able to adequately process all the information given. To avoid an overload of information, participants could freely choose which modules to visit, which steps to follow, and which assignments to make. Participants could stop anytime and continue at the point where they stopped. The use of video material might also be beneficial in the understanding of the information for survivors with low health literacy [99, 100]. Second, although the KNW is based on PST and CBT, there is no real patient-therapist interaction. Except by self-report, it is not possible to investigate whether the learned skills were applied in the right way [101]. In addition, it is difficult to anticipate the experienced emotions and non-verbal behavior of the participant or to give further explanation on why a certain advice is given. With computer tailoring, it is only possible to anticipate reactions that are highly expected. Third, while a greater proportion of cancer survivors are elderly [1], this group in general has fewer computer skills [102] and is less likely to use the Internet as a source for health-related information [24] than younger cancer survivors. To address this problem, the KNW was developed in such way that it is relatively easy to use. The invitation for participation was accompanied with a quick guide for using the KNW and the program provides instructional videos explaining how the KNW works. Also, support through telephone and e-mail is provided. It should be noted that this is only a temporary issue, as computer skills and use are increasing rapidly, especially among older adults [103].

In conclusion, the KNW is a theory and evidence based web-based computer tailored intervention that seems a promising tool to support cancer survivors to cope with cancer-related issues during life after treatment. The results of the RCT, which will be presented in other papers, will provide more insight into the effectiveness and working mechanisms of the KNW and its appreciation by its users.

## Abbreviations

KNW: Kanker Nazorg Wijzer (Cancer Aftercare Guide)
IM: Intervention Mapping
POs: Performance objectives
COs: Change objectives
PST: Problem-Solving Therapy
CBT: Cognitive Behavioral Therapy
RCT: Randomized Controlled Trial
